# Obligatory role for PKCδ in PIP_2_‐mediated activation of store‐operated TRPC1 channels in vascular smooth muscle cells

**DOI:** 10.1113/JP279947

**Published:** 2020-07-21

**Authors:** Miguel A. S. Martín‐Aragón Baudel, Jian Shi, William A. Large, Anthony P. Albert

**Affiliations:** ^1^ Department of Pharmacology University of California 451, Health Sciences Drive, Suite 3503 Davis CA USA; ^2^ Leeds Institute of Cardiovascular and Metabolic Medicine Faculty of Medicine and Health University of Leeds Leeds UK; ^3^ Vascular Biology Research Centre Molecular and Clinical Research Institute St George's University of London Cranmer Terrace London UK

**Keywords:** PKC, PIP, store‐operated channels_2_, TRPC1, vascular smooth muscle

## Abstract

**Key points:**

- In vascular smooth muscle cells (VSMCs), activation of Ca^2+^‐permeable store‐operated channels (SOCs) composed of canonical transient receptor potential channel 1 (TRPC1) subunits mediates Ca^2+^ entry pathways that regulate contraction, proliferation and migration, which are processes associated with vascular disease.
- Activation of TRPC1‐based SOCs requires protein kinase C (PKC) activity, which is proposed to phosphorylate TRPC1 proteins to promote channel opening by phosphatidylinositol 4,5‐bisphosphate (PIP_2_). We investigated the identity of the PKC isoform involved in activating TRPC1‐based SOCs in rat mesenteric artery VSMCs.
- TRPC1‐based SOCs were reduced by PKCδ inhibitors and knockdown of PKCδ expression. Store depletion induced interactions between TRPC1 and PKCδ and PKCδ‐dependent phosphorylation of TRPC1. Furthermore, generation of store‐operated interactions between PIP_2_ and TRPC1 and activation of TRPC1‐based SOCs by PIP_2_ required PKCδ.
- These findings reveal that PKCδ activity has an obligatory role in activating TRPC1‐based SOCs, through regulating PIP_2_‐mediated channel opening.

**Abstract:**

In vascular smooth muscle cells (VMSCs), stimulation of Ca^2+^‐permeable canonical transient receptor potential channel 1 (TRPC1)‐based store‐operated channels (SOCs) mediates Ca^2+^ entry pathways that regulate cell contraction, proliferation and migration, which are processes associated with vascular disease. It is therefore important to understand how TRPC1‐based SOCs are activated. Stimulation of TRPC1‐based SOCs requires protein kinase C (PKC) activity, with store‐operated PKC‐dependent phosphorylation of TRPC1 essential for channel opening by phosphatidylinositol 4,5‐bisphosphate (PIP_2_). Experimental protocols used to activate TRPC1‐based SOCs suggest that the PKC isoform involved requires diacylglycerol (DAG) but is Ca^2+^‐insensitive, which are characteristics of the novel group of PKC isoforms (δ, ε, η, θ). Hence, the present study examined whether a novel PKC isoform(s) is involved in activating TRPC1‐based SOCs in contractile rat mesenteric artery VSMCs. Store‐operated whole‐cell cation currents were blocked by Pico145, a highly selective and potent TRPC1/4/5 channel blocker and T1E3, a TRPC1 blocking antibody. PKCδ was expressed in VSMCs, and selective PKCδ inhibitory peptides and knockdown of PKCδ expression with morpholinos oligomers inhibited TRPC1‐based SOCs. TRPC1 and PKCδ interactions and phosphorylation of TRPC1 induced by store depletion were both reduced by pharmacological inhibition and PKCδ knockdown. In addition, store‐operated PIP_2_ and TRPC1 interactions were blocked by PKCδ inhibition, and PKCδ was required for PIP_2_‐mediated activation of TRPC1 currents. These results identify the involvement of PKCδ in stimulation of TRPC1‐based SOCs and highlight that store‐operated PKCδ activity is obligatory for channel opening by PIP_2_, the probable activating ligand.

## Key points

- In vascular smooth muscle cells (VSMCs), activation of Ca^2+^‐permeable store‐operated channels (SOCs) composed of canonical transient receptor potential channel 1 (TRPC1) subunits mediates Ca^2+^ entry pathways that regulate contraction, proliferation and migration, which are processes associated with vascular disease.
- Activation of TRPC1‐based SOCs requires protein kinase C (PKC) activity, which is proposed to phosphorylate TRPC1 proteins to promote channel opening by phosphatidylinositol 4,5‐bisphosphate (PIP_2_). We investigated the identity of the PKC isoform involved in activating TRPC1‐based SOCs in rat mesenteric artery VSMCs.
- TRPC1‐based SOCs were reduced by PKCδ inhibitors and knockdown of PKCδ expression. Store depletion induced interactions between TRPC1 and PKCδ and PKCδ‐dependent phosphorylation of TRPC1. Furthermore, generation of store‐operated interactions between PIP_2_ and TRPC1 and activation of TRPC1‐based SOCs by PIP_2_ required PKCδ.
- These findings reveal that PKCδ activity has an obligatory role in activating TRPC1‐based SOCs, through regulating PIP_2_‐mediated channel opening.

## Introduction

Store‐operated channels (SOCs) are Ca^2+^‐permeable plasmalemmal ion channels activated by depletion of cytosolic endo/sarcoplasmic (ER/SR) Ca^2+^ stores (Baudel *et al*. 2020). In vascular smooth muscle cells (VSMCs), SOCs are stimulated by vasoconstrictors that activate the Gαq protein‐coupled receptor signalling pathway leading to phospholipase C (PLC) activity, phosphatidylinositol 4,5‐bisphosphate (PIP_2_) hydrolysis, inositol 1,4,5‐trisphosphate (IP_3_) generation and IP_3_‐mediated depletion of SR Ca^2+^ stores. As such SOCs induce Ca^2+^ entry pathways that regulate vasoconstrictor‐mediated contraction, proliferation and migration, and are considered drug targets for treatment of vascular diseases such as hypertension and atherosclerosis.

There is substantial evidence that SOCs represent a diverse family of ion channels with differing molecular composition, activation mechanisms and functions (Albert & Large, 2003; Albert *et al*. 2007; 2009; Cheng *et al*. 2013; Prakriya & Lewis, 2015; Ong *et al*. 2015). In native contractile VSMCs, SOCs have relatively low Ca^2+^ permeability, linear current–voltage (*I*–*V*) rectification properties, a single channel conductance of 2–3 pS and are composed of canonical transient receptor potential (TRPC) channels (Trepakova *et al*. 2001; Xu & Beech, 2001; Albert & Large, 2002 *a*; Bergdahl *et al*. 2005; Liu *et al*. 2005 *a*,*b*; Xu *et al*. 2005 *a*; Albert *et al*. 2006; Saleh *et al*. 2006; 2008; 2009 *a*; Shi *et al*. 2012a, 2014, 2016, 2017a *a*). It is proposed that molecular composition involves a heteromeric TRPC1/C5 template with TRPC1 being the critical subunit for conferring activation by store depletion, hence the term TRPC1‐based SOCs (Xu & Beech, 2001; Xu *et al*. 2005 *a*; Saleh *et al*. 2008; Shi *et al*. 2012 *a*). In comparison, synthetic VSMCs that are involved in cell proliferation, migration and growth and are associated with more pathological functions exhibit TRPC1‐based SOCs and also SOCs with properties similar to Orai1‐based calcium release‐activated channels (CRACs) such as high Ca^2+^ permeability, pronounced inward rectification, single channel conductance in the fS range and composition of Orai1 proteins (Berra‐Romani *et al*. 2008; Beech, 2012; Trebak, 2012, Prakriya & Lewis, 2015). It is important to highlight that, in the present study, we examined activation mechanisms of TRPC1‐based SOCs in freshly isolated and primary cultured single VSMCs and tissue lysates that have a native contractile phenotype (Shi *et al*. 2016, 2017a *a*) and probably do not involve Orai1 proteins (Shi *et al*. 2017 *b*). These TRPC1‐based SOCs are probably important in regulating contractility and switching of VSMCs from contractile to synthetic phenotypes (Berra‐Romani *et al*. 2008; Matchkov *et al*. 2012).

How store depletion stimulates TRPC‐based SOCs is controversial, especially compared to Orai1‐based CRACs for which it is well‐established that store depletion induces the ER/SR store Ca^2+^ sensor stromal interaction molecule 1 (STIM1) to oligomerize and translocate to the plasma membrane where it interacts with Orai1 to induce channel assembly and gating (Prakriya & Lewis, 2015). However, there is growing evidence that store depletion also activates TRPC‐based SOCs through STIM1‐mediated processes, potentially involving direct interactions between TRPC and Orai1 proteins (Liao *et al*. 2014), activation of Orai1‐based CRACs as a prerequisite for TRPC1 channel opening (Ambudkar *et al*. 2017), and direct interactions between TRPC and STIM1 proteins (Worley *et al*. 2007; Yuan *et al*. 2009; Lee *et al*. 2014; Asanov *et al*. 2015). In native contractile VSMCs, our recent work indicates that store depletion stimulates TRPC1‐based SOCs through a novel STIM1‐mediated Gαq/PLCβ1 pathway, which probably induces channel opening by regulating interactions between protein kinase C and PIP_2_ (Shi *et al*. 2016, 2017a *a*).

There is considerable evidence that protein kinase C (PKC) activity is critical for activation of TRPC1‐based SOCs in native contractile VSMCs (Large *et al*. 2009; Albert, 2011). PKC inhibitors prevent activation of TRPC1‐based SOCs and reduce store‐operated phosphorylation of TRPC1, and PKC activators stimulate TRPC1‐based SOCs (Saleh *et al*. 2008, 2009a *a*; Shi *et al*. 2012a, 2016). Activation of TRPC1‐based SOCs and phosphorylation of TRPC1 by physiological vasoconstrictors are also reduced by PKC inhibitors (Albert & Large, 2002 *b*; Saleh *et al*. 2006; 2009 *b*; Shi *et al*. 2012b, 2014, 2016). It is proposed that PKC activity stimulates opening of TRPC1‐based SOCs through phosphorylation of TRPC1 proteins to promote PIP_2_ binding that acts as the activating ligand (Shi *et al*. 2014, 2016, 2017 *a*). As such, activation of TRPC1‐based SOCs by store depletion or PKC activators is inhibited by anti‐PIP_2_ antibodies and pre‐treatment with PI4‐kinase inhibitors which deplete PIP_2_ levels (Saleh *et al*. 2009 *a*; Shi *et al*. 2012 *b*). In addition, activation of TRPC1‐based SOCs by the water soluble PIP_2_ analogue diC8‐PIP_2_ is prevented by PKC inhibitors (Saleh *et al*. 2009 *a*; Shi *et al*. 2012 *b*). These results indicate that interactions between PKC activity and PIP_2_ have obligatory roles in activation of TRPC1‐based SOCs; PKC cannot activate TRPC1‐based SOCs without PIP_2_ and vice versa (Baudel *et al*. 2020). The present study further examines the importance of PKC activity in activating TRPC1‐based SOCs by investigating the PKC isoform(s) involved.

The PKC family comprises of at least 11 homologous serine/threonine kinases divided into three groups according to their basic structure and activation requirements: conventional PKC isoforms (α, βI, βII and γ) require both Ca^2+^ and diacylglyerol (DAG), novel PKC isoforms (δ, ε, η and θ) require DAG but are Ca^2+^‐insensitive, and atypical PKC isoforms (ζ, ι and λ) are activated by lipid mediators such as phosphatidylserine and do not require Ca^2+^ or DAG (Salamanca & Khalil, 2005; Ringvold & Khalil, 2017).

Many of these PKC isoforms are expressed in VSMCs and are proposed to regulate several physiological and pathological processes, including those reported to involve a role for TRPC1‐based SOCs (Salamanca & Khalil, 2005; Ding *et al*. 2011; Fan *et al*. 2014; Ringvold & Khalil, 2017). In native contractile VSMCs, stimulation of TRPC1‐based SOCs and PKC‐dependent phosphorylation of TRPC1 by store‐depletion requires PLCβ1 activity (Shi *et al*. 2016), DAG activates TRPC1‐based SOCs through a PKC‐dependent mechanism (Saleh *et al*. 2006; 2008; Large *et al*. 2009; Albert, 2011), and TRPC1‐based SOCs are activated by store depleting agents that probably increase (e.g. the SR Ca^2+^‐ATPase inhibitor cyclopiazonic acid), decrease (e.g. the cell‐impermeable and ‐permeable high affinity Ca^2+^ chelators BAPTA and BAPTA‐AM) or produce little change in [Ca^2+^]_i_ (e.g. the cell‐permeable low affinity Ca^2+^ chelator TPEN). These results suggest that the PKC isoform involved requires DAG, although it is probably Ca^2+^‐insensitive; these are characteristics of the novel group of PKC isoforms. These ideas on the identity of the PKC isoform involved form the basis of the present work, and our findings indicate that the PKCδ novel isoform is the probable candidate involved in activation of TRPC1‐based SOCs in native contractile rat mesenteric artery VSMCs.

## Methods

### Ethical approval

All animal procedures were carried out in accordance with guidelines laid down by St George's, University of London Animal Welfare Committee, and conform with the principles and regulations described by the Service Project Licence: 70/8512, and also to the principles and regulations of *The Journal of Physiology* as described by Grundy (2015). Male Wistar rats (8–12 weeks old) were used for the purpose of the present study. Rats were supplied from Charles River (Margate, UK) and housed and maintained in standard sized plastic cages at the Biological Research Facility at St George's, University of London, under a 12:12 h light/dark photocycle, at 18–20 °C and ∼50% relative humidity, with water and laboratory rodent diet (Specialist Dietary Services, UK) available *ad libitum*. Animals were culled by cervical dislocation in accordance with the UK Animals Scientific Procedures Act of 1986 and as revised by European Directive 2010/63/EU.

### Cell isolation and tissue lysates

Mesenteric arteries were dissected and cleaned of adherent fat in physiological salt solution containing (mmol L^−1^): 126 NaCl, 6 KCl, 10 glucose, 11 Hepes, 1.2 mgCl_2_, and 1.5 CaCl_2_ with pH adjusted to 7.2 with 10 mol L^−1^ NaOH. Single VSMCs were enzymatically dispersed and tissue lysates prepared as described previously (Shi *et al*. 2016, 2017a *a*).

### PKCδ knockdown

Knockdown of PKCδ was performed by transfection of vessel segments with morpholino anti‐sense oligonucleotides as described previously (Stott *et al*. 2018). PKCδ morpholino oligomers and a control oligomer containing five mismatched nucleotides (10 μm; Genetools, Philomath, OR, USA) were mixed with lipofectamine 2000 (Life Technologies, Carlsbad, CA, USA) in Opti‐MEM and left at room temperature for 2 h. Mesenteric artery segments were cultured with this mix in Dulbecco's modified Eagle's medium F‐12 with 1% penicillin/streptomycin at 37°C for 48 h. Vessel segments were then enzymatically dispersed into single VSMCs or used as tissue lysates as required.

### Electrophysiology

Whole‐cell and cell‐attached patch clamp techniques were used to record TRPC1‐based SOCs with an Axopatch 200B amplifier (Axon Instruments, Union City, CA, USA) at room temperature (20–23°C) using bath and patch pipette solutions, data analysis and experimental protocols as described previously (Shi *et al*. 2017 *a*). In experiments investigating the effects of phorbol 12,13‐dibutyrate, a phorbol ester and PKC activator, and diC8‐PIP_2_ on whole‐cell cation currents, 750 ms duration voltage ramps from +100 mV to −150 mV were applied every 30 s from a holding potential of 0 mV. The patch pipette solution contained (mm): 126 CsCl, 1.2 MgCl_2_, 10 nHepes, 11 glucose, 10 BAPTA, 4.8 CaCl_2_, ∼100 nm free internal Ca^2+^ concentration as calculated using MaxChelator (https://somapp.ucdmc.ucdavis.edu/pharmacology/bers/maxchelator), 1 Na_2_ATP, 0.2. NaGTP, pH 7.2 with Tris. The external solution contained (mm): 126 NaCl, 1,5 CaCl_2_, 10 Hepes, 11 glucose, 0.1. DIDS, 0.1 niflumic acid and 0.005 nicardipine, pH 7.2 with NaOH.

### Immunoprecipitation and western blotting

Freshly isolated or cultured vessel segments were prepared for immunoprecipitation, one‐dimensional protein gel electrophoresis and immunoblotting as described previously (Shi *et al*. 2017 *a*). The primary antibodies used were: mouse anti‐PKCε (dilution 1:500; sc‐1681; Santa Cruz Biotechnology, Santa Cruz, CA, USA), mouse anti‐PKCθ (dilution 1:500; sc‐1680; Santa Cruz Biotechnology), mouse anti‐PKCη (dilution 1:500; sc‐136 036; Santa Cruz Biotechnology) and rabbit anti‐PKCδ (dilution 1:1000; ab182126; Abcam, Cambridge, MA, USA). Rabbit anti‐TRPC1 antibody (1 μg mL^−1^) was generated by GenScript (Piscataway, NJ, USA) using peptide sequences from a previously characterized putative extracellular region (Xu & Beech, 2001). Visualization were performed using anti‐rabbit and anti‐mouse secondary antibodies conjugated to IRDye 800RD or IRDye 680CW (dilution 1:10 000; Li‐Cor Biosciences, Cambridge, UK) as appropriate and the Odyssey Infrared Imaging System (Li‐Cor Biosciences). Protein band intensities were measured using Image Studio software (Li‐Cor Biosciences) and normalized to smooth muscle actin (dilution 1:2000; #ab5694; Abcam) when quantified.

### Immunofluorescence

Single VSMCs were fixed with 4% (w/v) paraformaldehyde for 15 min, cells were treated with 0.1 mol L^−1^ glycine for 5 min and permeabilized with phosphate‐buffered saline (PBS) containing 0.1% (v/v) Triton X‐100 (PBS‐T) for 15 min at room temperature. Cells were then incubated with PBS‐T containing 1% (w/v) BSA for 1 h at room temperature to block non‐specific binding of antibodies. Immunostaining was performed with mouse anti‐PKCε (dilution 1:500; sc‐1681; Santa Cruz Biotechnology), mouse anti‐PKCθ (dilution 1:200; sc‐1680; Santa Cruz Biotechnology), rabbit anti‐PKCη (dilution 1: 200; ab4134; Abcam), rabbit anti‐PKCδ (dilution 1:1000; ab182126; Abcam) overnight at 4°C. Cells were then washed and incubated with a 488 fluorophore‐conjugated donkey anti‐goat secondary antibody (dilution 1:1000; A‐11 055; Alexa Fluor; Thermo Fisher Scientific, Waltham, MA, USA) for 1 h at room temperature. Unbound secondary antibodies were removed by washing with PBS, and nuclei were labelled with 4′,6‐diamidino‐2‐phenylindole mounting medium (Sigma, Poole, UK). Control experiments were performed by omitting either primary or secondary antibodies. Cells were imaged using an A1R confocal microscope (Nikon, Tokyo, Japan). PIP_2_ immunofluorescence experiments were performed in the presence of saponin with rabbit anti‐PIP_2_ (sc‐53 412, 1:50; Santa Cruz Biotechnology) as described previously (Edimo *et al*. 2016).

### Proximity ligation assay

Interactions between different proteins were investigated with a Duolink® *in situ* detection kit (Sigma) as described previously (Shi *et al*. 2017 *a*). Single VSMCs were fixed and permeabilized as per immunofluorescence and blocking was performed with Duolink blocking buffer for 1 h at 37°C. Cells were then incubated with appropriate antibodies overnight at 4°C as per immunofluorescence experiments plus mouse anti‐P‐Ser (dilution 1:100; sc‐16B4; Santa Cruz Biotechnology), mouse anti‐P‐Thr (dilution 1:100; sc‐5267; Santa Cruz Biotechnology), mouse anti‐TRPC1 (dilution 1:100; sc‐133 076; Santa Cruz Biotechnology) and rabbit anti‐TRPC5 (T5E3), which was generated by GenScript using peptide sequences from a previously characterized putative extracellular region (1 μg mL^−1^) ((Xu & Beech, 2001; Xu *et al*. 2005a, 2005b *a*,*b*). The rest of the protocol was conducted in accordance with the manufacturer's instructions. Fluorescent puncta were visualized with an A1R confocal microscope and images were analysed with ImageJ Fiji (https://fiji.sc). The mean number of puncta per cell was calculated by counting the number of particles across a z‐stack of the cell.

### Reagents

Pico145 was a generous gift provided by Robin S Bon and David J Beech (University of Leeds, Leeds, UK). The cell permeable PKCδ inhibitor, δV1‐TAT peptide (RRRQRRKKRGY‐SFNSYELGSL) was synthesized by Mimotopes (Wirral, UK). PKCδ non‐permeable δPKC_8‐17_ peptide inhibitor (SFNSYELGSL) was purchased from AnaSpec (AnaSpec, EGT Corporate Headquarters, Fremont, CA, USA). PKCε peptide inhibitor was purchased from Santa Cruz Biotechnology. All other drugs were purchased from Sigma or Tocris (Abingdon, UK). Agents were dissolved in distilled H_2_O or 0.1% DMSO. DMSO alone had no effect on whole‐cell or single channel currents.

### Statistical analysis

All statistical analysis was performed using Prism, version 8 (GraphPad Software Inc., San Diego, CA, USA). Data were calculated as the mean ± SD, with *n* indicating the number of data points. Data points were generated from at least three different isolated VSMCs or tissue lysate preparations. To compare between two or more *I*–*V* relationships, two‐way ANOVA with Tukey's multiple comparisons test was used, and differences in means at −80 mV are reported. To compare between two data sets, paired or unpaired *t* tests were used. *P* < 0.05 was considered statistically significant.

## Results

### PKC‐dependent store‐operated currents are composed of TRPC1 subunits in rat mesenteric artery VSMCs

**Figure 1:**
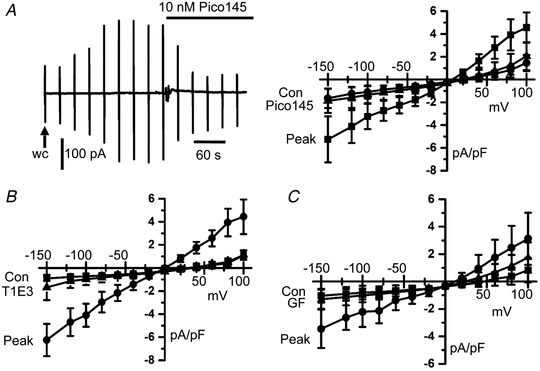
TRPC1 compose PKC‐dependent SOCs in native contractile VSMCs *A*, representative trace and mean *I*–*V* relationships showing that the development of store‐operated whole‐cell currents from control to peak levels in freshly isolated rat mesenteric artery VSMCs following obtaining whole‐cell configuration (wc) was inhibited by bath application of Pico145. Vertical deflections represent currents evoked by voltage ramps from +100 mV to −150 mV (750 ms duration) every 30 s from a holding potential of 0 mV. *B* and *C*, Mean data showing that bath applications of T1E3 (1 μg mL^−1^) and GF109203X (3 μm) inhibited store‐operated whole‐cell currents.

In our first series of experiments, we confirmed that PKC‐dependent TRPC1‐based SOCs are functionally expressed in native contractile VSMCs from freshly isolated rat mesenteric arteries using a highly selective and potent TRPC1/C4/C5 channel blocker Pico145 (Rubaiy *et al*. 2017), an externally‐acting TRPC1 blocking antibody T1E3 (Xu & Beech, 2001, Xu *et al*. 2005 *b*) and the pan‐PKC isoform selective inhibitor GF109203X. Figure 1*A* shows that passive depletion of internal Ca^2+^ stores, following cell dialysis with a patch pipette solution containing high concentrations of BAPTA and no added Ca^2+^, induced whole‐cell cation currents that had a relatively linear *I*–*V* relationship, an *E*_rev_ between 0 mV and +20 mV, with a mean control amplitude of −1.15 ± 0.62 pA pF^–1^ at −80 mV which increased to a mean peak amplitude of −2.74 ± 0.88 pA pF^–1^ (*n* = 6, *p* = 0.0015) and reduced to −0.89 ± 0.35 pA pF^–1^ by bath application of Pico145 (*n* = 6, *p* = 0.0002, two‐way ANOVA, Tukey's multiple comparisons test). Figure 1*B* and *C* show that bath application of T1E3 and G109203X also inhibited store‐operated whole‐cell currents, with mean peak amplitudes at −80 mV reduced respectively from −2.96 ± 0.92 pA pF^–1^ to −0.84 ± 0.43 pA pF^–1^ (*n* = 6, *p* = 0.0001) and from −2.14 ± 0.92 pA pF^–1^ to −0.91 ± 0.45 pA pF^–1^ (*n* = 7, *p* = 0.0051, two‐way ANOVA, Tukey's multiple comparisons test).

**Figure 2:**
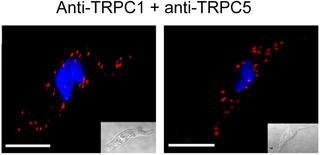
Expression of TRPC1‐TRPC5 interactions in native contractile VSMCs Representative PLA images from two different freshly isolated rat mesenteric artery VSMCs showing the association between TRPC1 and TRPC5. These images, and other PLA images, represent puncta observed across a z‐stack of each cell. Scale bar = 10 μm.

There is considerable evidence that heteromeric TRPC1/C5 molecular structures compose TRPC1‐based SOCs in VSMCs from different rabbit, mouse and human vascular beds (see Introduction). In proximity ligation assay (PLA) studies, Figure 2 shows that fluorescent puncta with a mean number per cell of 51.5 ± 12.6 (*n* = 14) produced using anti‐TRPC1 and anti‐TRPC5 antibodies were present at (or close to) the plasma membrane in freshly isolated rat mesenteric artery VSMCs.

These findings clearly confirm that PKC‐dependent TRPC1‐based SOCs are functionally expressed in native contractile rat mesenteric artery VSMCs, similar to previous studies in VSMCs from different rabbit, mouse and human vascular preparations (see Introduction).

### PKCδ is the dominant novel PKC isoform in VSMCs

The PKC isoform involved in activating TRPC1‐based SOCs in VSMCs requires DAG but is Ca^2+^ insensitive and is probably a member of the novel PKC isoform family (see Introduction). We therefore investigated the expression of novel PKC isoforms in tissue lysates from freshly isolated rat mesenteric arteries. We used brain lysates as positive controls where novel PKC isoforms have been previously identified (Fleegal *et al*. 2005; Popp *et al*. 2006; Callender & Newton, 2017; Wang *et al*. 2019; Yang *et al*. 2019).

**Figure 3:**
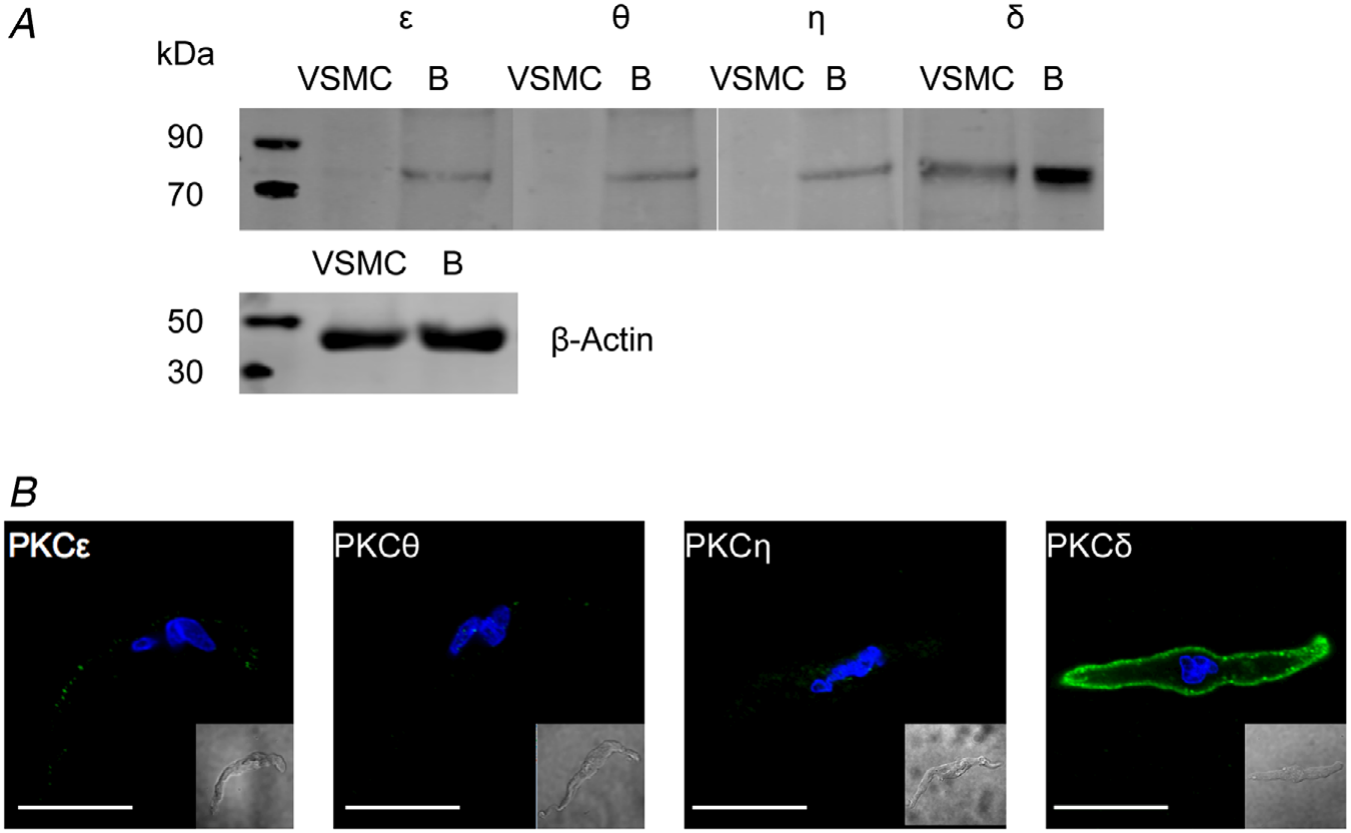
Expression of novel PKC isoforms in native contractile VSMCs *A*, representative western blots showing expression of the novel PKC isoforms PKCε, PKCθ, PKCη and PKCδ in rat mesenteric artery VSMCs and brain (*B*) protein lysates. *B*, representative immunocytochemical images of rat mesenteric VSMCs immunolabelled with the same anti‐novel PKC isoform antibodies used in (*A*). Scale bar = 10 μm.

Figure 3 *a* shows PKCδ expression with relatively low levels or little expression of PKCε, PKCη and PKCθ were found in tissue lysates from rat mesenteric arteries. Using the same anti‐PKC novel isoform antibodies, expression of PKCδ, PKCε, PKCη and PKCθ was present in brain lysates. In addition, Figure 3 *b* shows that immunocytochemical studies revealed PKCδ staining at (or close to) the plasma membrane of VSMCs with little staining recorded for PKCε, PKCη and PKCθ isoforms.

### PKCδ activity is essential for activation of TRPC1‐based SOCs

In our next series of experiments, we examined whether pharmacological inhibition of PKCδ and knockdown of PKCδ expression using morpholino technology resulted in an anticipated decrease in activation of TRPC1‐based SOCs. We focused on the PKCδ isoform based on the expression studies detailed above and previous data on the expression and function of PKCδ in vascular smooth muscle (Salamanca & Khalil, 2005; Ringvold & Khalil, 2017).

**Figure 4:**
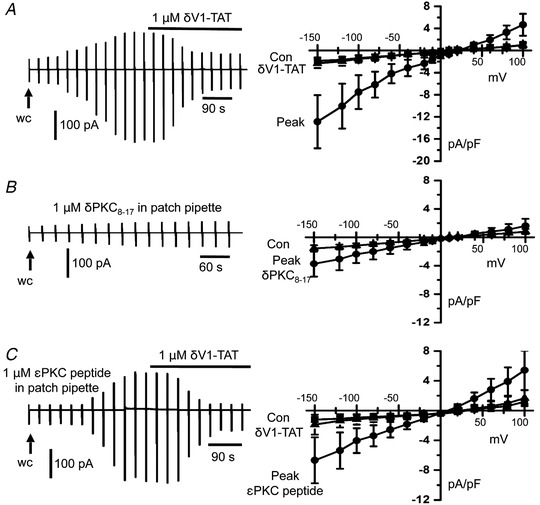
Pharmacological PKCδ inhibitors reduce TRPC1‐based SOCs *A*, representative trace and mean *I*–*V* relationship showing that the peak amplitude of store‐operated whole‐cell TRPC1 currents in freshly isolated rat mesenteric artery VSMCs was reduced by bath application of δV1‐TAT. *B*, a small store‐operated whole‐cell TRPC1 current was induced following inclusion of δPKC_8‐17_ peptide inhibitor in the patch pipette solution. *C*, inclusion of εPKC peptide inhibitor in the patch pipette appeared to have little effect on the development of store‐operated whole‐cell TRPC1 currents, which were subsequently inhibited by bath application of δV1‐TAT.

Figure 4*A* reveals that store‐operated whole‐cell TRPC1‐based currents were inhibited by bath application of the cell‐permeable PKCδ inhibitor δV1‐TAT, with mean peak amplitude reduced from −6.18 ± 2.34 pA pF^–1^ to −1.26 ± 0.57 pA pF^–1^ at −80 mV (*n* = 6, *p* = 0.0001, two‐way ANOVA, Tukey's multiple comparisons test). Figure 4*B* also shows that following inclusion of the cell‐impermeable PKCδ peptide inhibitor δPKC_8‐17_ in the patch pipette solution store depletion induced a small whole‐cell TRPC1‐based current with mean control amplitude increasing from −0.97 ± 0.27 pA pF^–1^ to a mean peak amplitude of −2.01 ± 1.1 pA pF^–1^ at −80 mV (*n* = 6, *p* = 0.0902, two‐way ANOVA, Tukey's multiple comparisons test).

Because expression of PKCε was also observed in VSMCs (Fig. 3) and other studies have reported the expression of PKCε in the vasculature (Salamanca & Khalil, 2005; Ringvold & Khalil, 2017), we investigated the potential contribution of this PKC isoform in activating store‐operated TRPC1‐based SOCs. Figure 4*C* shows that, following inclusion of a PKCε‐specific peptide inhibitor in the patch pipette solution, store depletion activated whole‐cell TRPC1 currents with a mean control amplitude increasing from −0.84 ± 0.21 pA pF^–1^ to −3.39 ± 1.42 pA pF^–1^ at −80 mV (*n* = 5, *p* = 0.0006), which was subsequently inhibited by bath application of δV1‐TAT to −0.99 ± 0.56 pA pF^–1^ (*n* = 5, *p* = 0.0012, two‐way ANOVA, Tukey's multiple comparisons test).

**Figure 5:**
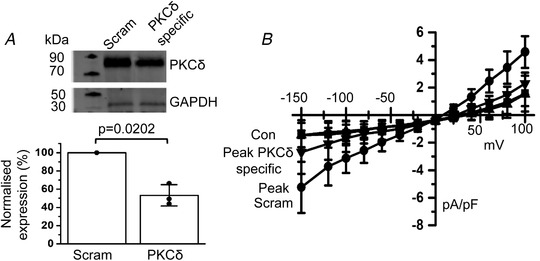
Knockdown of PKCδ expression reduces activation of TRPC1‐based SOCs *A*, representative western blots and mean data showing that PKCδ‐specific morpholino oligomers reduced PKCδ expression in rat mesenteric artery protein lysates compared to scrambled/mismatched oligomers (Scram). Band intensity was normalized to β‐GAPDH to calculate percentage expression (*vs*. scrambled, *n* = 3, *p* = 0.0202, unpaired *t* test). *B*, mean *I*–*V* relationships showing that the mean peak amplitude of store‐operated whole‐cell TRPC1 currents was reduced in the presence of PKCδ‐specific (*n* = 10) compared to scrambled (*n* = 9) morpholino oligomers.

Figure 5*A* shows that incubation of freshly isolated rat mesenteric artery segments with anti‐sense morpholino oligomers against PKCδ for 48 h significantly decreased protein expression of PKCδ by ∼50% compared to a mismatched oligomer (*n* = 3, *p* = 0.0202, unpaired *t* test). Figure 5*B* shows that, in VSMCs isolated from vessel segments expressing mismatched (scrambled) oligomers, store depletion induced whole‐cell TRPC1 currents with relative linear rectification and a mean control amplitude at −80 mV that increased from −1.07 ± 0.68 pA pF^–1^ to a mean peak amplitude of −2.57 ± 0.89 pA pF^–1^ (*n* = 9, *p* = 0.0003, two‐way ANOVA, Tukey's multiple comparisons test), which were similar to currents recorded from freshly isolated VSMCs (Figs 1 and 4). By contrast, Figure 5*B* shows that, in VSMCs expressing specific PKCδ oligomers, store depletion activated small whole‐cell TRPC1 currents, with the control mean amplitude at −80 mV increasing from −1.05 ± 0.59 pA pF^–1^ to a mean peak amplitude of −1.36 ± 0.69 pA pF^–1^ (*n* = 10, *p* = 0.8240, two‐way ANOVA, Tukey's comparisons test). As such, mean peak amplitudes of store‐operated TRPC1 current at −80 mV were greatly reduced in VSMCs expressing specific PKCδ compared to scrambled oligomers (*p* = 0.0051, two‐way ANOVA, Tukey's comparisons test).

### Store depletion induces TRPC1 and PKCδ interactions and PKCδ‐dependent phosphorylation of TRPC1

The above pharmacological and molecular evidence indicates that PKCδ activity is essential for activation of TRPC1‐based SOCs in native contractile VSMCs. Therefore, we investigated whether store depletion induced PKCδ and TRPC1 interactions and PKCδ‐dependent phosphorylation of TRPC1 in freshly isolated rat mesenteric artery tissue lysates and VSMCs.

**Figure 6:**
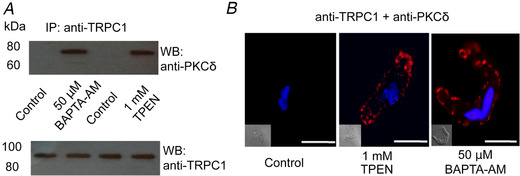
Store depletion induces associations between TRPC1 and PKCδ in native contractile VSMCs *A*, representative western blots with anti‐TRPC1 and anti‐PKCδ antibodies following immunoprecipitation of freshly isolated rat mesenteric artery tissue lysates with an anti‐TRPC1 antibody show that pre‐treatment with the BAPTA‐AM and TPEN induced associations between TRPC1 and PKCδ but did not alter TRPC1 expression. *B*, representative images of proximity ligation assays from freshly isolated rat mesenteric artery VSMCs showing a low level of association between TRPC1 and PKCδ in unstimulated VSMCs, which was greatly increased following pre‐treatment with TPEN and BAPTA‐AM for 10 min. Scale bars = 10 μm.

Immunoprecipitation with anti‐TRPC1 antibodies followed by immunoblotting with an anti‐PKCδ antibody revealed that interactions between these two proteins are absent in unstimulated tissue but occur following incubation with BAPTA‐AM or TPEN (Fig. 6*A*). Furthermore, the PLA studies shown in Figure 6*B* confirmed that interactions between TRPC1 and PKCδ were absent in unstimulated VSMCs, whereasd robust puncta at (or close to) the plasma membrane were induced between TRPC1 and PKCδ following pre‐treatment with BAPTA‐AM and TPEN.

**Figure 7:**
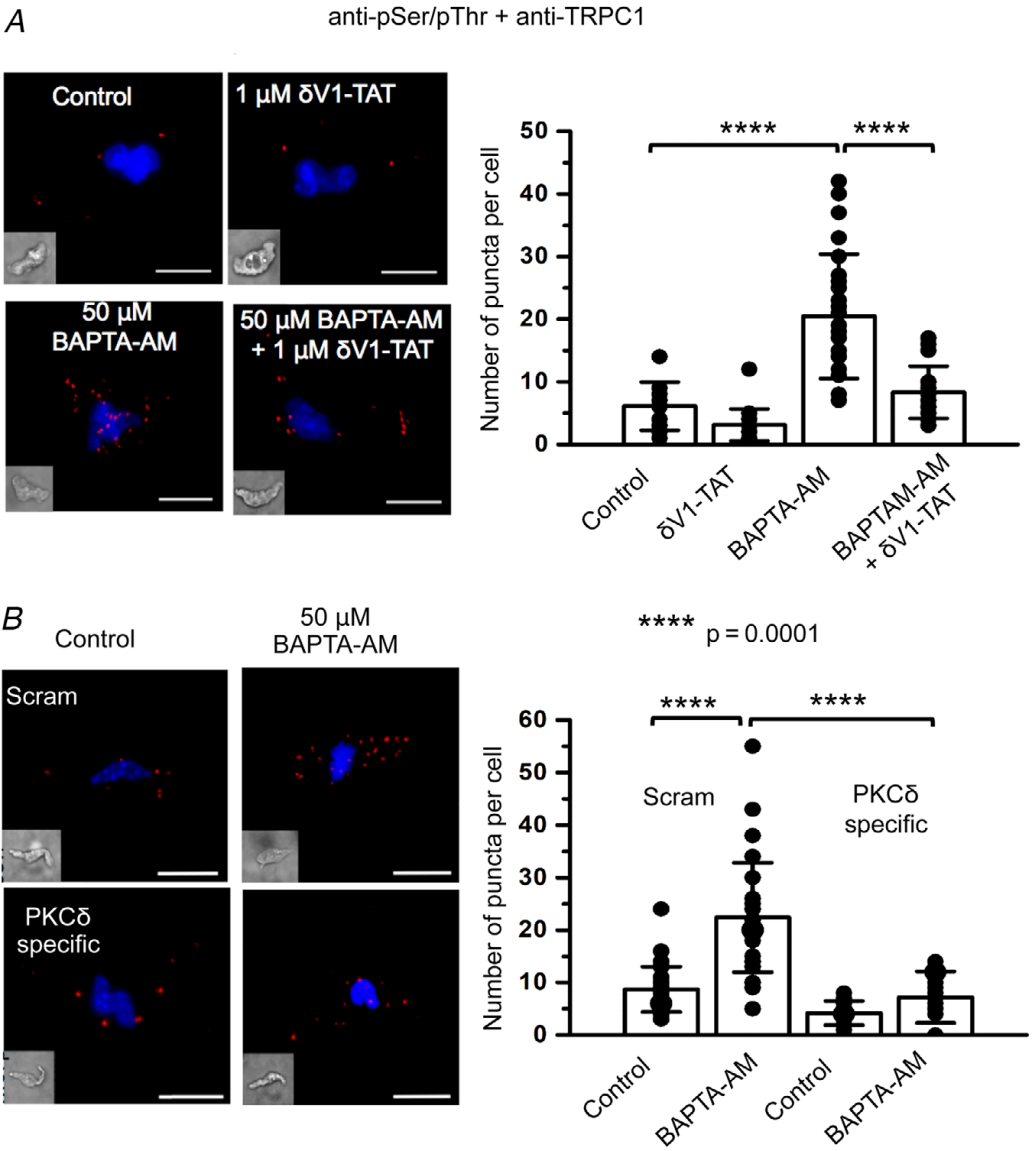
Store depletion induces PKCδ‐dependent phosphorylation of TRPC1 *A*, representative and mean data from proximity ligation assays showing pre‐treatment with BAPTA‐AM (*n* = 30) for 10 min greatly increased the number of puncta per cell representing associations between TRPC1 and phosphorylated serine and threonine residues compared to control values (*n* = 19, *p* = 0.0001), which was reduced in the presence of δV1‐TAT (*n* = 25, *p* = 0.0001, unpaired *t* test). *B*, representative and mean data from proximity ligation assays showing that BAPTA‐AM‐induced associations between TRPC1 and phosphorylated serine and threonine residues were reduced in VSMCs from vessels transfected with PKCδ‐specific (*n* = 24, *p* = 0.0001) compared to scrambled morpholino oligomers (*n* = 30, *p* = 0.0001, unpaired *t* test). Scale bars = 10 μm. ^****^ *p* = 0.0001.

PLA experiments performed with a mixture of anti‐phosphorylated serine/threonine and anti‐TRPC1 antibodies revealed a low number of fluorescent puncta in unstimulated freshly isolated VSMCs, which is indicative of a low level of basal TRPC1 phosphorylation. Figure 7*A* shows that the mean number of puncta per cell was increased following pre‐treatment with BAPTA‐AM from 6.1 ± 3.9 (*n* = 19) to 20.5 ± 9.9 (*n* = 30, *p* = 0.0001) and inhibited by coapplication of δV1‐TAT to 8.3 ± 4.2 (*n* = 25, *p* = 0.0001, unpaired *t* test). In addition, Figure 7*B* shows that transfection of vessel segments with specific PKCδ morpholino oligomers also reduced the BAPTA‐AM‐induced increase in mean puncta number compared to scrambled oligomers from 22.4 ± 10.4 (*n* = 30) to 7.2 ± 4.9 (*n* = 25, *p* = 0.0001, unpaired *t* test). Importantly, Figure 7*A* and *B* shows a similar mean number of puncta using mismatched oligomers compared to non‐transfected freshly isolated VSMCs under unstimulated (8.7 ± 4.3, *n* = 26 *vs*. 6.1 ± 3.9, *n* = 19, *p* = 0.049) or BAPTA‐AM‐treated conditions (22.4 ± 10.4, *n* = 30 *vs*. 20.5 ± 9.9, *n* = 30, *p* = 0.4653, unpaired *t* test), indicating that the transfection process had little effect on this mechanism.

Taken together, these results indicate that store depletion induces interactions between TRPC1 and PKCδ in VSMCs, and that these interactions cause PKCδ‐dependent phosphorylation of TRPC1.

### PKCδ mediates store‐operated interactions between PIP_2_ and TRPC1, as well as PIP_2_‐evoked TRPC1 currents

In our previous work, we identified that PIP_2_ has an obligatory role in the activation of TRPC1‐based SOCs in VSMCs, potentially as the activating ligand, and that this process requires PKC‐dependent phosphorylation of TRPC1 (see Introduction). Therefore, we investigated whether store‐operated interactions between PIP_2_ and TRPC1 require PKCδ activity using immunocytochemistry and PLA, and also whether PKCδ is essential for activation of store‐operated whole‐cell TRPC1 currents by PIP_2_.

**Figure 8:**
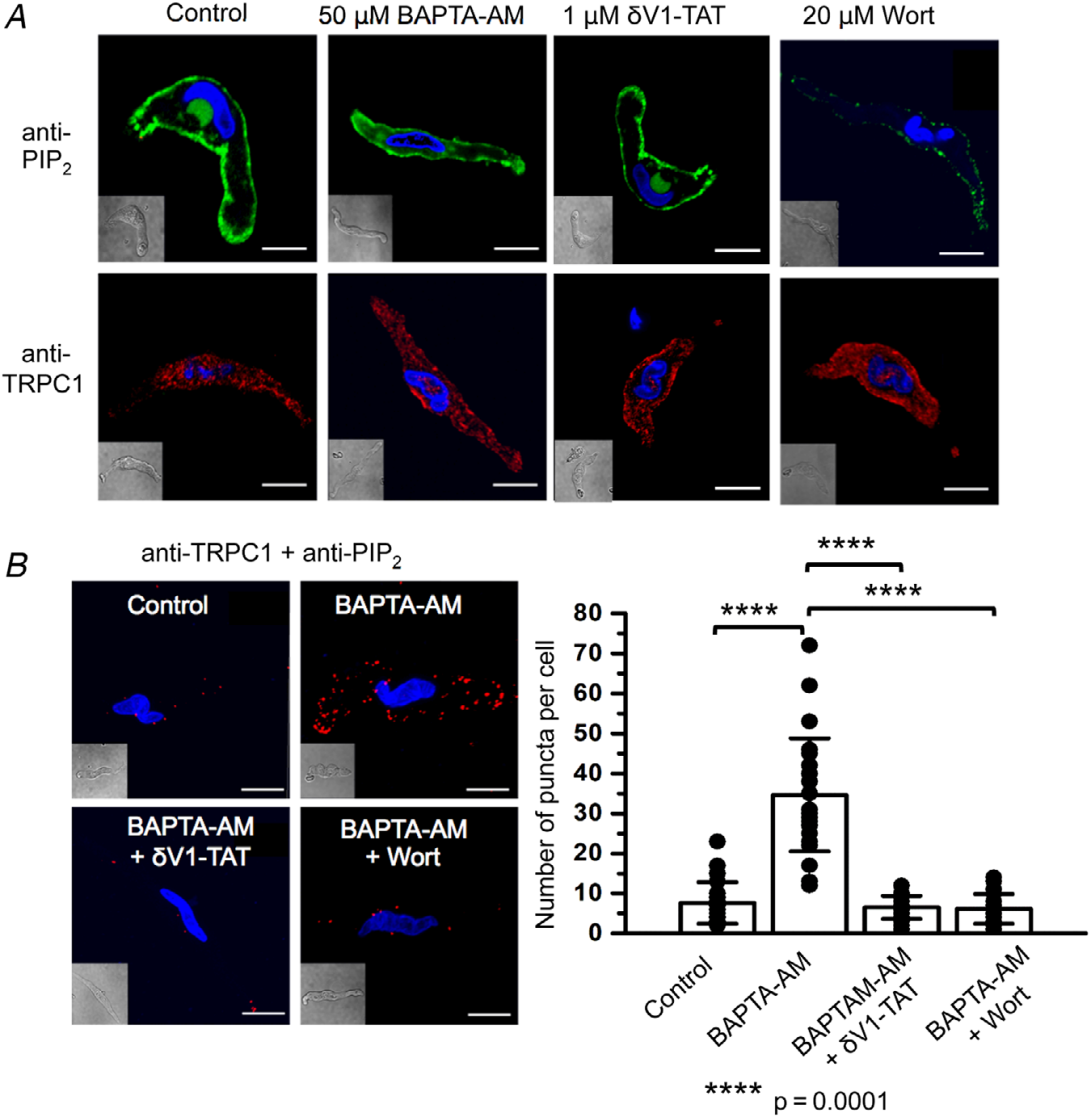
Store‐operated interactions between TRPC1 and PIP_2_ require PKCδ *A*, representative images from freshly isolated rat mesenteric VSMCs showing expression of PIP_2_ (green) and TRPC1 (red) following pretreatment with BAPTA‐AM for 10 min, wortmannin (Wort) for 20 min and δV1‐TAT for 10 min. *B*, representative images and mean data from PLA experiments showing that control interactions between TRPC1 and PIP_2_ (*n* = 23) were greatly increased by pre‐treatment with BAPTA‐AM for 10 min (*n* = 23, *p* = 0.0001), and this was reduced by coapplication with δV1‐TAT (*n* = 20, *p* = 0.0001) and wortmannin (*n* = 19, *p* = 0.0001, unpaired *t* test). Scale bars = 10 μm.

In freshly isolated rat mesenteric artery VSMCs, Figure 8*A* shows that PIP_2_ immunostaining was highly expressed at (or close to) the plasma membrane in unstimulated cells and BAPTA‐AM and δV1‐TAT had no obvious effect on this distribution. With respect to the staining produced with anti‐PIP_2_ antibodies being related to endogenous PIP_2_, Figure 8*A* shows that pre‐treatment with high concentrations of wortmannin (20 μm), which inhibits PI‐4/PI‐5 kinases to reduce PIP_2_ recycling and promote PIP_2_ depletion, produced a considerable reduction in the PIP_2_ signal (Suh & Hille, 2005; Saleh *et al*. 2009 *a*; Shi *et al*. 2014). In control experiments, TRPC1 immunostaining was unaffected by BAPTA‐AM, δV1‐TAT or wortmannin treatment (Fig. 8*A*). We next adapted this immunostaining protocol for PLA experiments. Figure 8*B* reveals that there was a low level of interactions between PIP_2_ and TRPC1 in unstimulated VSMCs, which were increased by BAPTA‐AM from a mean puncta number per cell of 7.6 ± 5.2 (*n* = 23) to 34.6 ± 14.1 (*n* = 24, *p* = 0.0001) and inhibited in the presence of δV1‐TAT or following pre‐treatment of wortmannin, respectively, to 6.5 ± 2.9 (*n* = 20, *p* = 0.0001) and 6.1 ± 3.7 (*n* = 19, *p* = 0.0001, unpaired *t* test).

**Figure 9:**
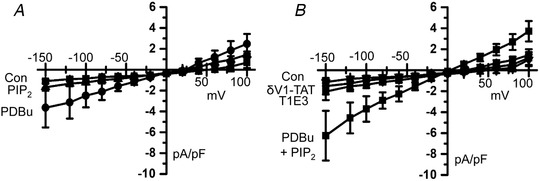
PIP_2_ increases PDBu‐induced whole‐cell TRPC1 currents *A*, mean *I*–*V* relationships from freshly isolated rat mesenteric artery VSMCs showing that bath application of PDBu but not inclusion of diC8‐PIP_2_ in the patch pipette solution induced a whole‐cell current. *B*, mean *I*–*V* relationships showing that, following cell dialysis with diC8‐PIP_2_, PDBu‐evoked whole‐cell currents had a greatly increased peak amplitude compared to currents induced by PDBu alone (*A*) and were inhibited by T1E3 and δV1‐TAT.

In freshly isolated VSMCs, Figure 9*A* shows that bath application of the phorbol ester 12,13‐dibutyrate (PDBu) increased whole‐cell non‐selective cation currents from a mean control amplitude of −0.71 ± 0.13 pA pF^–1^ to a mean peak amplitude of −2.12 ± 0.8 pA pF^–1^ at −80 mV (*n* = 8, *p* = 0.0001, two‐way ANOVA, Tukey's comparisons test). By contrast, inclusion of diC8‐PIP_2_ in the patch pipette solution had no effect on whole‐cell cation currents (−80 mV, −0.75 ± 0.18 *vs*. −1.0 ± 0.08, *n* = 5, *p* = 0.6151, two‐way ANOVA, Tukey's comparisons test). Figure 9*B* shows that dialysing cells with diC8‐PIP_2_ in the patch pipette solution increased PDBu‐activated whole‐cell cation currents, with mean peak amplitude at −80 mV increased from −2.12 ± 0.8 pA pF^–1^ (*n* = 8) (Fig. 9*A*) to −2.88 ± 0.78 pA pF^–1^ at −80 mV (*n* = 7, *p* = 0.0291, unpaired *t* test). Figure 9*B* also shows that PDBu‐evoked whole‐cell cation currents induced in the presence of diC8‐PIP_2_ were inhibited by bath applications of T1E3 and δV1‐TAT, respectively, to −1.13 ± 0.24 pA pF^–1^ (*n* = 5, *p* = 0.0001) and −0.84 ± 0.38 pA pF^–1^ at −80 mV (*n* = 7, *p* = 0.0001 two‐way ANOVA, Tukey's comparisons test) indicating that these currents were mediated by TRPC1 and required PKCδ activity.

### Noradrenaline‐evoked TRPC1 channels require PKCδ

**Figure 10:**
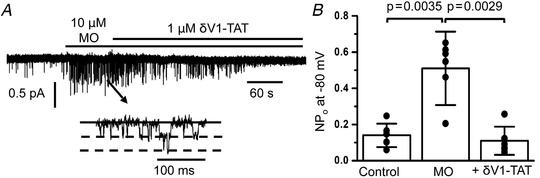
Methoxamine‐evoked TRPC1 channel activity is mediated by PKCδ *A* and *B*, representative trace and mean open probability (NP_o_) data showing that bath application of methoxamine evoked TRPC1 channel activity in cell‐attached patches held at −80 mV (*n* = 6, *p* = 0.0035), which was reduced by coapplication of δV1‐TAT (*n* = 6, *p* = 0.0029, paired *t* test).

The present study clearly demonstrates that the PKCδ isoform is important for activation of TRPC1 channels by agents that deplete internal Ca^2+^ stores. Therefore, in the final series of experiments, we investigated the physiological significance of these findings by examining whether PKCδ is involved in activation of TRPC1 channels by the vasoconstrictor and α_1_‐adrenoceptor agonist methoxamine (MO). Figure 10 shows that, in unstimulated VSMCs, there was a low level of spontaneous 2 pS TRPC1‐based channel activity in cell‐attached patches held at −80 mV, and that mean peak open probability (NP_o_) was increased following bath application of MO from 0.14 ± 0.07 to 0.51 ± 0.2 (*n* = 6, *p* = 0.0035) and was significantly reduced by subsequent coapplication of δV1‐TAT to 0.11 ± 0.08 (*n* = 6, *p* = 0.0029, paired *t* test).

## Discussion

The present study reveals that activity of PKCδ, a member of the novel PKC isoform subgroup, is essential for activation of TRPC1‐based channels evoked by store depletion and α_1_‐adrenoceptor stimulation in native contractile rat mesenteric artery VSMCs. It is proposed that PKCδ‐dependent phosphorylation of TRPC1 is required for TRPC1 channel activation by PIP_2_, the probable activating ligand. These results provide further evidence that PKC activity and PIP_2_ are obligatory for activation of TRPC1‐based SOCs in VSMCs, ion channels implicated in regulating contraction, proliferation and migration that are processes linked to hypertension and atherosclerosis. As such, regulating PKCδ activity and PIP_2_ levels may comprise therapeutic targets for these and other vascular diseases.

### TRPC1‐based SOCs require PKC activity

Our results show that the well‐established protocol for inducing store depletion, the inclusion of a high concentration of BAPTA in the patch pipette solution, evoked a whole‐cell non‐selective cation current with a relatively linear *I*–*V* relationship and an *E*_rev_ of between 0 mV and +20 mV in freshly isolated rat mesenteric artery VSMCs, which is similar to currents previously recorded in rabbit and mouse VSMCs (Shi *et al*. 2012a, 2014, 2016, 2017a, 2017b *a,b*). Considerable evidence obtained using pharmacological agents, antibodies as blocking agents and TRPC1 knockout mice indicates that these store‐operated currents are mediated by a heteromeric TRPC1/TRPC5 molecular template (Xu & Beech, 2001; Xu *et al*. 2005 *a*; Saleh *et al*. 2008; Shi *et al*. 2012 *a*). In further support of this proposal, the present study shows that store‐operated whole‐cell currents were inhibited by Pico145, a highly selective and potent TRPC1/C4/C5 channel blocker (Rubaiy *et al*. 2017) and T1E3, a TRPC1 antibody raised against an extracellular pore region and known to act as a blocking agent (Xu & Beech, 2001; Xu *et al*. 2005 *b*). In addition, using PLA, the present study reveals that TRPC1 and TRPC5 co‐localize within <40 nm of each other at (or close to) the plasma membrane of VSMCs. It should be noted that the electrophysiological and pharmacological properties of these store‐operated currents in freshly isolated VSMCs exhibiting a contractile phenotype are considerably different from those of Orai1‐based CRACs (Prakriya & Lewis, 2015), indicating that Orai1 proteins are probably not involved, as reported previously (Shi *et al*. 2017 *b*).

Previous studies show that PKC activity and PKC‐mediated phosphorylation of TRPC1 are essential for activation of TRPC1‐based SOCs in native contractile VSMCs (Albert & Large, 2002 *b*; Saleh *et al*. 2006, 2008; Large *et al*. 2009; Albert, 2011; Shi *et al*. 2012a, 2014, 2016). The present study provides further support for an excitatory role for PKC, with the pan‐PKC isoform selective inhibitor GF109203X inhibiting store‐operated whole‐cell TRPC1 currents in freshly isolated rat mesenteric artery VSMCs. Therefore, identifying the specific PKC isoform responsible for activating TRPC1‐based SOCs was the main aim of the present study.

### PKCδ is the PKC isoform responsible for activation of TRPC1‐based SOCs and store‐operated TRPC1 phosphorylation

The PKC family is subdivided into conventional PKC (α, β and γ), novel PKC (δ, ϵ, η and θ) and atypical PKC (ζ and ι/κ) isoforms (Salamanca & Khalil, 2005; Ringvold & Khalil, 2017). Given that DAG analogues activate TRPC1 channels through a PKC‐dependent mechanism in VSMCs (Saleh *et al*. 2006; 2008; Large *et al*. 2009; Albert, 2011), and that the Ca^2+^ store depletion protocols used to activate TRPC1‐based SOCs probably increase, decrease or do not alter [Ca^2+^]_i_, the PKC isoform(s) responsible is probably belongs to the novel subfamily (see Introduction).

Western blotting and immunocytochemical analysis demonstrated that PKCδ is expressed in native contractile rat mesenteric artery VSMCs and was distributed at (or close to) the plasma membrane. There was greater expression of PKCδ compared to other novel PKC isoforms in VSMCs and tissue lysates; however, caution is warranted when suggesting that PKCδ is the dominant novel PKC isoform in VSMCs because expression variations may be linked to potential differences between the affinity and specificity of the anti‐novel PKC isoform antibodies used.

Inhibition of PKCδ activity via well‐characterized selective PKCδ peptide inhibitors and knockdown of PKCδ expression using morpholino oligomers resulted in a significant reduction in the development and peak amplitude of store‐operated whole‐cell TRPC1 currents. Moreover, immunoprecipitation and PLA studies revealed that store depletion induced interactions between TRPC1 and PKCδ. Importantly, PLA experiments showed that store depletion induced puncta formation between anti‐TRPC1 and anti‐phosphorylated serine and threonine antibodies that was greatly reduced by PKCδ peptide inhibitors and PKCδ selective morpholino oligomers, which indicates that store depletion induces PKCδ‐dependent phosphorylation of TRPC1. Taken together, these findings provide strong evidence for the PKCδ isoform being essential in the activation of TRPC1‐based SOCs in native contractile VSMCs.

PKCδ has also been reported to participate in store‐operated Ca^2+^ entry in airway smooth muscle (Gao *et al*. 2012), proliferation and migration of VSMCs (Ding *et al*. 2011; Fan *et al*. 2014), development of the myogenic response (Kashihara *et al*. 2008), and membrane insertion of TRPM4 channels and associated vasoconstriction (Crnich *et al*. 2010; Garcia *et al*. 2011). Interestingly, TRPC1 has also been associated with many of these vascular functions (Earley & Brayden, 2015) and, accordingly, in the future, it may be useful to investigate the functional association between TRPC1 and PKCδ activities in the vasculature. By contrast to the results outlined above, PKC activity has an inhibitory action on Orai1‐based CRACs with PKC inhibitors including GF109203X and knockdown of PKCβ isoform increasing Orai1 activity (Kawasaki *et al*. 2010), which further indicates that Orai1 proteins or Orai1‐based CRACs are not involved in the activation of TRPC1‐based SOCs in native contractile VSMCs.

### PKCδ activity is required for PIP_2_‐mediated activation of TRPC1 channels

We have previously described the obligatory requirement of PIP_2_ in the activation of TRPC1‐based SOCs and proposed that PKC‐dependent phosphorylation of TRPC1 is required for this phospholipid to act as the activating ligand (Large *et al*. 2009; Saleh *et al*. 2009a, 2009b *a*,*b*; Albert, 2011; Shi *et al*. 2012 *a*,2012b, 2014). In the present study, using PLA, store depletion induces interactions between PIP_2_ and TRPC1 at (or close to) the plasma membrane of VSMCs that are reduced by PKCδ peptide inhibitor. To provide evidence that endogenous PIP_2_ is involved in these results, we showed that a high concentration of wortmannin (20 μm) that depletes PIP_2_ levels by inhibiting PI‐4/PI‐5 kinase‐mediated PIP_2_ synthesis (Suh & Hille, 2005) reduced PIP_2_ immunostaining and store‐operated associations between TRPC1 and PIP_2_. It should be noted that a high concentration of wortmannin also inhibits myosin light chain kinase (MLCK) and PI(3) kinase, although previous findings have shown that lower concentrations (<1 μm) inhibiting MLCK and PI‐3 kinase but not depleting PIP_2_ levels had no effect on activation of TRPC1 channel activity in VSMCs (Saleh *et al*. 2009 *a*; Shi *et al*. 2014). The PKC activator PDBu activated whole‐cell TRPC1 currents that were increased in amplitude when the water soluble PIP_2_ analogue diC8‐PIP_2_ was included in the patch pipette solution and were inhibited by a PKCδ peptide inhibitor. Cell dialysis with diC8‐PIP_2_ failed to activate any whole‐cell currents on its own, as reported previously (Albert *et al*. 2008), which indicates that the effects of PDBu and PIP_2_ are not merely additive but probably represent synergism between the two molecules. Taken together, these results re‐enforce the idea that PIP_2_ is the activating ligand of TRPC1‐based SOCs, and that this requires PKCδ‐dependent phosphorylation of TRPC1.

The proposed excitatory roles of PKC and PIP_2_ on TRPC1‐based SOCs are opposite to the action of these molecules in the activation of non‐TRPC1‐containing channels (e.g. TRPC3/C6/C7 channels) in native contractile VSMCs (Venkatachalam *et al*. 2003; Albert & Large, 2004; Large *et al*. 2009; Shi *et al*. 2010; Albert, 2011). This subgroup of TRPC channels, known as receptor‐operated channels (ROCs), is activated by receptor‐mediated generation of DAG, which leads to channel opening via PKC independent mechanisms, with subsequent PKC activity induced by DAG producing channel inhibition. Interestingly, stimulation of TRPC1‐based SOCs is proposed to inhibit TRPC6‐based SOCs through inducing Ca^2+^ influx and PKC activity, which suggests the involvement of a conventional PKC isoform (Shi *et al*. 2010). A distinct role of PIP_2_ on TRPC3/C6/C7‐based ROCs is less clear, with both inhibitory and excitatory actions on channel activity being proposed (Albert *et al*. 2008; Imai *et al*. 2012). These findings further indicate that TRPC1‐based SOCs and TRPC3/C6/C7‐based ROCs form distinct channel structures with differing activation mechanisms involving differing PKC isoforms, and probably distinct functions in VSMCs.

Computation predication of potential PKCδ‐dependent phosphorylation sites within the TRPC1 sequence using GPS 3.0 (Xue *et al*. 2008) reveals five intracellular serine residues, with Ser619 and Ser752 at the C‐terminal domain being of significance in that both of these sites are close to a known PIP_2_‐binding domain (Kwon *et al*. 2007). Hence, it is possible that PKCδ‐dependent phosphorylation of one or both sites increases PIP_2_ binding to TRPC1, leading to channel opening. Other studies have demonstrated that protein kinase A, protein kinase G and calmodulin kinase II have inhibitory actions on TRPC1‐based SOCs in VSMCs (Liu *et al*. 2005 *a*; Albert *et al*. 2006; Chen *et al*. 2011), and therefore TRPC1 phosphorylation by these kinases might inhibit TRPC1‐based SOCs by reducing TRPC1 and PIP_2_ interactions. Similar roles for kinase activities with respect to modulating lipid–protein interactions are well‐established in the regulation of K^+^ channel subtypes (Logothetis *et al*. 2015).

One important aspect is how PKCδ activity is stimulated by store depletion. Our proposed hypothesis based on previous work indicates that store depletion induces STIM1–TRPC1 interactions, which stimulate a Gq‐PLCβ1 pathway that generates DAG and PKC activity, leading to PKC‐dependent phosphorylation of TRPC1 (Baudel, 2020). PKC‐dependent phosphorylation of TRPC1 leads to a dissociation of interactions between TRPC1 and the PIP_2_ binding protein myristoylated alanine‐rich C‐kinase substrate (MARCKS), with the latter molecule then releasing PIP_2_ into the local environment, allowing it to act as the channel activating ligand. The present study indicates that the PKCδ isoform is probably an essential PKC isoform in this process and this will be important to investigate in detail in future studies.

### Physiological relevance of PKCδ‐mediated TRPC1 channels

TRPC1 channel activity induced by the α_1_‐adrenoceptor agonist and vasoconstrictor methoxamine was reduced by a PKCδ peptide inhibitor, which suggests that a similar store‐operated activation process involving PKCδ may be used by physiological stimulants of TRPC1 channels. This is further supported by findings showing that PLCβ1 and STIM1 knockdown both significantly inhibit noradrenaline‐evoked TRPC1 channel activity (Shi *et al*. 2016, 2017b *b*) and noradrenaline induces an increase in PKC‐dependent phosphorylation of TRPC1 and stimulates MARCKS to release PIP_2_, which is then available to interact with TRPC1 (Shi *et al*. 2014). It was previously proposed that vasoconstrictor agents may also activate TRPC1‐based SOCs independently of store deletion (Albert & Large, 2002 *b*; Saleh *et al*. 2006; Large *et al*. 2009; Saleh *et al*. 2009 *b*; Shi *et al*. 2010, 2012b *b*; Albert, 2011), which suggests that TRPC1 channels may also act as ROCs. The current and previous findings certainly suggest that a significant contribution to physiological stimulation of TRPC1 channels probably occurs via a store‐operated pathway.

## Additional information

### Competing interests

The authors declare that they have no competing interests.

### Author contributions

MMAB, JS, WL and AA all contributed to the conception or design of the work, as well as the analysis of data or the interpretation of data for the work, and were involved in drafting the work or revising it critically for important intellectual content. MMAB and JS were involved in the acquisition of data. All authors approved final version of the manuscript submitted for publication and agree to be accountable for all aspects of the work. All persons designated as authors qualify for authorship, and all those who qualify for authorship are listed.

### Funding

This work was supported by the Biotechnology and Biological Sciences Research Council (BB/J007226/1 and BB/M018350/1 to AA).

## Supporting information


**Statistical Summary Document**

## Data Availability

The data that support the findings of this study are available from the corresponding author upon reasonable request.
